# HPV Testing by cobas HPV Test in a Population from Catalonia

**DOI:** 10.1371/journal.pone.0058153

**Published:** 2013-03-06

**Authors:** Belén Lloveras, Silvia Gomez, Francesc Alameda, Beatriz Bellosillo, Sergi Mojal, Mercè Muset, Manuel Parra, José Carlos Palomares, Sergi Serrano

**Affiliations:** 1 Department of Pathology, Hospital del Mar, Universitat Autònoma de Barcelona, Barcelona, Spain; 2 Consulting Service on Methodology for Biomedical Research, IMIM, Barcelona, Spain; 3 Clinical Unit of Infectious Diseases and Microbiology, Hospital de Valme, Sevilla, Spain; Baylor College of Medicine, United States of America

## Abstract

**Background:**

HPV testing in cervical cancer screening has been proposed as an alternative or complementary to cytology in women older than 30 years. However, adequate clinical sensitivity and specificity are crucial for a new test to be implemented. Hybrid Capture 2 (HC2) has proved good clinical performance in selecting women at risk for high-grade intraepithelial lesions with a high sensitivity and specificity. cobas HPV Test has been recently launched and its performance in different clinical settings needs to be determined.

**Objectives:**

The aim of this study was to evaluate the cobas HPV Test for the detection of cervical HPV infection in a population of women in Catalonia (Spain) using HC2 as a reference.

**Materials and Methods:**

Cervical liquid cytology samples from 958 women have been studied. Sensitivity was analyzed in 60 samples from patients with a high-grade intraepithelial lesion (≥CIN2) on histology and specificity was determined in 898 samples from women with no ≥CIN2. All cases had HC2 and cobas HPV Test performed. Statistical analyses of sensitivity, specificity and comparison between HC2 and cobas HPV Test by a non-inferiority test were applied.

**Results:**

Sensitivity of HC2 and cobas HPV Test for detecting ≥CIN2 proved identical (98.3%) while specificity was 85.3% and 86.2% respectively. The non-inferiority test demonstrated that cobas HPV Test surpassed 90% sensitivity and 98% specificity of HC2.

**Conclusion:**

The cobas HPV Test results fulfilled sensitivity and specificity requirements for HPV based cervical cancer screening and for the triage of minor cytological abnormalities, allowing its introduction in clinical settings.

## Background

High-risk human papillomavirus (HR-HPV) have been demonstrated as the causal agents of cervical cancer [1], [2]. HPV detection has been proposed as an alternative or complementary to cytology, according to a better sensitivity for the detection of precancerous lesions, so-called high-grade cervical intraepithelial lesions (CIN 2 or CIN 3) and cancer in cervical cancer screening [3]–[9]. However, HPV based techniques harbour different analytical sensitivities and more important, if specificity is too low, a large number of women without lesions would be derived for colposcopy [10].

Hybrid capture 2 (HC2, Qiagen, Hilden, Germany), the most widespread HPV test, is considered to have an adequate clinical sensitivity with an acceptable specificity [4]–[11] and it was the first test approved in the triage of Atypical Squamous Cells of Unknown Significance (ASC-US) by the FDA (www.fda.gov). Some studies have shown that the risk for CIN2 or worse (≥CIN2) at 3–5 years after a negative HPV test is 40–50% lower than after a negative cytology due to its high negative predictive value [3], [7], [9], [12], [13].

Recently other HPV tests have been developed with limited information on clinical performance. Foreseeing the situation, guidelines were published by a group of experts to clinically validate HPV tests against the gold standard HPV test HC2, mainly focusing on the detection of ≥CIN2 in women ≥30 years old. A new candidate test should detect ≥CIN2 with sensitivity not less than 90% and specificity at least of 98% compared to HC2 [14].

## Objectives

The main objective of our study was to evaluate the cobas HPV Test from Roche Molecular System (Pleasanton, California, USA) in the detection of ≥CIN2 in comparison to the results of HC2 in opportunistic screening for cervical cancer in Catalonia, Spain.

## Materials and Methods

The project was approved by the PSMar Ethics Committee (CEIC-PSMAR 2010/4009/I). Samples were obtained from clinical diagnosis material. After diagnosis, part of the material was anonymized and collected for future research projects. The CEIC-PSMAR approved the use of these samples for the project (waived the need of specific informed consent).

The Pathology department of the Hospital del Mar (Parc de Salut MAR Biobanc) is the reference laboratory for cervical cytology in an area with a population of approximately 150.000 women, of which 105.000 are 30 years or older. From a total of 2700 consecutive samples from September 2009 until October 2010 that had HC2 performed, 958 that fulfilled the eligibility criteria were included in the study. One group, consisting of 898 cases was used for the evaluation of HPV test specificity. These cases corresponded to women with the following inclusion criteria: ≥30 years, no abnormal cytology in Hospital del Mar in the previous 5 years, minimum of 10 ml of PreservCyt cell suspension. A second group, consisting of 60 samples, was used to determine the sensitivity of the HPV test for detecting intraepithelial high-grade lesions. All 60 cases corresponded to histologically confirmed ≥CIN2 selected, including 22 CIN 2 and 38 CIN 3. The only inclusion criteria for these cases was a minimum amount of 10 ml of PreservCyt cell suspension, with no limits on age or past history of abnormal cytology.

The samples were collected in a ThinPrep vial with 30 ml of PreservCyt (Hologic, Marlborough, MA, U.S.A.) and a slide for cytologic study was produced first. This procedure is based on a system that aspirates the cell suspension sample through a filter and stops aspiration when an adequate number of epithelial cells are obtained, therefore depending on the amount of cellularity the sample that remains is variable. Experienced cytotechnologists performed the screening with assistance of the automated system Imager (Hologic) and the results were reported according to the Bethesda system [16]. The remaining material from the PreservCyt cellular sample was used for HC2 and cobas HPV Test if 10 ml or more were still available.

Intralaboratory reproducibility was assessed in 546 samples and consisted of performance by a different technician. The microbiology laboratory in Hospital de Valme, Sevilla, Spain was responsible for the testing of 445 samples to evaluate interlaboratory reproducibility, using as a reference the results from the first test at Hospital del Mar. The smaller sample size in this part of the study was due to consumption of some samples.

HC2 is based on hybridization with RNA probes to detect a total of 13 HR-HPV (16,18, 31, 33, 35, 39, 45, 51, 52, 56, 58, 59 and 68) and signal amplification. First, PreservCyt samples must be converted to be suitable for HC2. In short, 6ml are centrifuged with a conversion buffer and then the pellet is resuspended in denaturing reagent and incubated for 45 minutes at 65°C. Following denaturation, hybridization is performed on a microtiter plate mixing 75 µl of the sample and 25 µl of a cocktail of RNA probes for 60 minutes at 65°C. Then the samples and controls are transferred to another microtiter plate with anti-hybrid antibodies and incubated for 60 minutes. Signal amplification is based on peroxidase-labelled anti-hybrid antibodies. The final step consists on adding a substrate that gives a chemiluminiscent signal that is measured by a luminometer. The analytical sensitivity of the test is set at 1 pg of HPV DNA/ml by the positive controls. The result is reported as positive when the semi-quantitative value (RLU: Relative Light Units), that is the ratio between each sample RLU, and the mean RLU of 3 positive controls, is ≥1.

The cobas HPV Test, recently approved by the FDA, is a fully automated test based on a simultaneous extraction of the HPV and cellular DNA, followed by a real-time PCR designed to detect 14 high risk HPVs using LightCycler®480 technology. Analytical sensitivity measured with different amounts of SiHa and HeLa cells in PreservCyt solution, according to the kit insert, is 100 copies/ml for HPV 16 and 40 copies/ml for HPV 18. In short, 400 µl of each sample in Preservcyt are directly processed from the original vial with a minimum amount of 3 ml for DNA extraction with the X 480 system. Denaturation is achieved with high temperature and chaotropic agents. DNA is purified by magnetic beads system and a microtiter plate with the samples is generated. The plate is then introduced in the z 480 real-time PCR system. HPV 16 and 18 are detected separately and other 12 HR-HPV types (31, 33, 35, 39, 45, 51, 52, 56, 58, 59, 66 and 68) are detected as a pool by a cocktail of probes with 3 different fluorochromes. In the same reaction the human beta-globin gene is amplified and detected by a different fluorochrome as a control for the entire process. A positive and a negative control are also included. The results are expressed as negative or positive for each HPV 16, HPV 18 or other HR-HPVs. When neither beta-globin nor HPV is detected the result is invalid. The value of cycle threshold (Ct) in quantitative PCR inversely correlates with the amount of target DNA present in the sample, in this case HPV DNA. Ct of each sample is not displayed in the results sheet but it is easily accessible in the system.

To estimate the clinical sensitivity and specificity conventional contingency tables were used, calculating percentages and their 95% confidence intervals. Cobas HPV Test sensitivity and specificity for ≥CIN2 were compared to HC2 by a non-inferiority score test, according to the suggested relative sensitivity for ≥CIN2 of at least 90% and relative specificity of ≥98% [14]. Agreement between tests was assessed by Cohen’s kappa statistics and the minimum value considered by guidelines, indicating good agreement is 0.7.

A Mann-Whitney U test was performed to compare RLU and Ct positive values between discordant vs. concordant results and diagnostic categories. Intraclass correlation coefficient (ICC) was applied to assess reproducibility of Cts in the intralaboratory study. In all analysis a p value less than 0.05 was considered statistically significant. Analyses were performed with SPSS 15.0 (SPSS Inc, Chicago, Il., U.S.A.).

## Results

The mean age of all the women included in the study was 46.78 years (range 23–89). Patients with ≥CIN2 had a mean age of 34.93 (range 23–63).

A valid cobas HPV Test assay was obtained in all samples tested except for one case that was excluded, showing both negative beta-globin and HPV results. HC2 and cobas HPV Test sensitivities were identical (98.3%; 95% confidence interval (CI): 95.1–100), missing a different ≥CIN2 each. Among <CIN2 cases there were 54 cases HPV positive by one method only, of these 31 were HPV positive by HC2 and 23 by cobas HPV Test. Specificity was 85.3% (95% C.I.: 83–87.6) for HC2 and 86.2% (95% C.I.: 83.9–88.4) for cobas HPV Test. Agreement between both tests including all samples was 94.2% with a kappa value of 0.814 (Table 1).

**Table 1:** Sensitivity and specificity of HC2 and cobas HPV Test.

		cobas HPV Test −	cobas HPV Test +	Total
**<CIN2**	HC2 −	743 (82.7%)	23 (2.6%)	766 (85.3%)
	HC2+	31 (3.5%)	101 (11.2%)	132 (14.7%)
	Total	774 (86.2%)	124 (13.8%)	898 (100%)
**≥CIN2**	HC2 −	0 (0%)	1 (1.6%)	1 (1.6%)
	HC2+	1 (1.6%)	58 (96.7%)	59 (98.4%)
	Total	1 (1.6%)	59 (98.4%)	60 (100%)

HC2 sensitivity for ≥ CIN2: 98.3% (95% CI: 95.1–100); cobas HPV test sensitivity for ≥ CIN2: 98.3% (95% CI: 95.1–100); HC2 specificity for ≥ CIN2: 85.3% (95% CI: 83–87.6); cobas HPV Test specificity for ≥ CIN2: 86.2% (95% CI: 83.9–88.4).

The analysis of sensitivity and specificity for ≥CIN2 for cobas HPV Test compared to the gold standard HC2 by a non-inferiority test showed that the candidate test was not inferior (relative sensitivity of 90%, T = 2.354, *P* = 0.009 and relative specificity of 98%, T = 3.045, *P* = 0.001).

The HPV genotype results for cobas HPV Test showed HPV 16 in 31≥CIN2, in 11 CIN 1, in 2 ASC-US and in 16 normal cytology samples. HPV 18 was detected in 4≥CIN2 (in 2 as the only type), in 2 CIN 1 and in 8 negative cytology samples. Other HR-HPV were detected in 34≥CIN2 (26 cases without HPV 16 or 18), 29 LSIL, 3 ASC-US and 71 cytology negative samples.

Regarding intralaboratory reproducibility for the HPV negative/positive results, kappa was 0.957. Only 9 out of 546 samples showed discordant results (Table 2). Type-specific reproducibility showed kappa values for HPV 16, HPV 18 and other HR-HPV types of 0.960, 0.956 and 0.949 respectively.

**Table 2:** Intralaboratory reproducibility.

	1st cobas HPV Test		
2nd cobas HPV Test	Negative	Positive	Total
Negative	372 (68.1%)	6 (1.1%)	378 (69.2%)
Positive	3 (0.6%)	165 (30.2%)	168 (30.8%)
Total	375 (68.7%)	171 (31.3%)	546 (100%)

Overall test agreement (HR-HPV Positive *vs.* Negative) *kappa value = *0.957.

Interlaboratory reproducibility showed a kappa value of 0.971 for an HPV positive/negative result, with only 5 discordant results (Table 3). HPV type reproducibility kappa values were 0.987, 1 and 0.943 for HPV 16, HPV 18 and other HR-HPV respectively.

**Table 3:** Interlaboratory reproducibility between Hospital del Mar (first test) and Hospital de Valme.

	cobas HPV Test HMar (1^st^ HPV test)		
cobas HPV Test HValme	Negative	Positive	Total
Negative	324 (72.8%)	2 (0.4%)	326 (73.2%)
Positive	3 (0.7%)	116 (26.1%)	119 (26.8%)
Total	327 (73.5%)	118 (26.5%)	445 (100%)

Overall test agreement (HR-HPV Positive *vs.* Negative) *kappa value* = 0.971.

As a measure for viral load reproducibility, intraclass correlation coefficient (ICC) between Cts of the intralaboratory study depicted high values: 0.995 for HPV 16, 0.947 for HPV 18 and 0.984 for HR-HPVs. ICCs were higher for Cts below 35 than for Cts over 35.

The comparison of semi-quantitative viral load values of HC2 and cobas HPV Test from women with or without ≥CIN2 showed statistically significant differences in HC2 RLU and cobas HPV Test Ct values. That is to say that ≥CIN2 samples with an HPV positive result, showed higher mean viral loads by both methods, with the exception of samples harbouring HPV 16 or HPV 18, where no differences according to cytological diagnosis were found (Table 4).

**Table 4:** Mean, standard deviation, range, median and IQR of HC2 RLU and cobas HPV test Ct according to diagnosis, in HPV positive cases.

Diagnosis	HC2+ RLU	HPV 16+Ct	HPV 18+Ct	HR-HPV+Ct	HC2+ RLU
**< CIN2**	N	132	29	10	103
	Mean (SD)	275.05 (529.14)	30.03 (5.98)	32.39 (5.1)	31.38 (5.56)
	Range	1.03–2466.43	21–40	26–39	17.4–39.8
	Median[IQR]	21.00[228.6]	28.00 [9.8]	32.80 [9.7]	31.90 [9.8]
**≥ CIN2**	N	59	31	4	34
	Mean (SD)	717.39 (888.11)	26.63 (2.9)	30.88 (7.14)	26.75 (4.56)
	Range	1.46–4189.08	20–32	22–37	18.7–37
	Median[IQR]	336.96[1095]	26.90 [4.4]	32.60 [13.1]	27.20 [7.5]
Statistical analysis		P<0.001	n.s.	n.s.	p<0.001

Discordant results accounted for 32 HC2 positive/cobas HPV Test negative and 24 cobas HPV Test positive/HC2 negative cases. Cases with concordant HPV positive results showed statistically significant higher viral loads than cases with discordant results (Table 5).

**Table 5:** Mean, standard deviation, range, median and IQR of HC2 RLU and cobas HPV Test Ct for concordant (A) and discordant (B) HC2 and cobas HPV Test results.

A	HC2+cobas HPV test +				
	RLU	Ct HPV 16		Ct HPV 18	Ct HR-HPV
N	159	55		11	119
Mean (SD)	491.67 (730.48)	27.36 (4.022)		30.35 (5.07)	29.09 (5.17)
Range	1.03–4189.08	20–40		22–37	17.4–39.5
Median (IQR)	150.95 (692.89)	26.9 (5.2)		28.7 (10.1)	28.6 (7.3)
**B**	**HC2+cobas HPV test −**	**HC2– cobas HPV test +**			
	**RLU**	**Ct HPV 16**		**Ct HPV 18**	**Ct HR-HPV**
N	32	5		3	18
Mean (SD)	14.25 (27.68)	38.3 (1.375)		37.87 (1.22)	37.82 (1.48)
Range	1.06–128.96	36–40		36.8–39.2	34.3–39.8
Median (IQR)	2.19 (5.61)	38.3 (2.4)		37.6 (2.1)	38.1 (1.78)
Statistical analysis	p<0.001	p<0.001		p = 0.005	p<0.001

## Discussion

The introduction of HPV testing in cervical cancer screening based on a better sensitivity compared to cytology has the risk of an excessive number of women without relevant lesions being referred for colposcopy. It is important to remark that the key issue is to detect HPV infections that may reveal ≥CIN2 on biopsy, avoiding the clinically irrelevant infections. The high negative predictive value of HC2 has been proved useful in long term studies not only in triaging women with minor cytological abnormalities but also in screening women over the age of 30, whose risk of an HPV infection being persistent is higher than in younger women [17], [18]. According to European and U.S. meta-analyses the relative sensitivity and specificity of HC2 compared to cytology are 1.43 and 0.96, however, the type of HPV test, its analytical sensitivity but most crucially its clinical sensitivity and specificity are decisive factors before its introduction as a screening tool [18]. As technology evolves and molecular techniques are being implemented in many clinical laboratories there is a need for validation of new tests. Roche Molecular Systems (Pleasanton, Ca.) has developed the cobas HPV Test based in real-time PCR that includes automated DNA extraction. Its ability to perform 282 tests per day with only 8 hours of hands-on time makes the system very attractive for busy clinical laboratories. We aimed to evaluate its applicability in the Catalan opportunistic screening protocol, which presently is based on cytology and HC2.

In this series of consecutive cases, the cobas HPV test system showed excellent results regarding both sensitivity and specificity. Non-inferiority test analysis consolidates it as being at least as good as HC2, the present gold standard in HPV testing in cervical samples. Both intralaboratory and interlaboratory reproducibility kappa values were in the category considered almost perfect, overcoming the minimal requirements for a clinical test. Recently, other groups have published similar results in other settings, one of them being the ATHENA trial including 1578 women with ASC-US that was used for the FDA approval of cobas HPV Test [15], [19].

HPV genotyping by cobas HPV Test showed more HPV 16 among ≥CIN2 than in other diagnostic categories, supporting the idea that it is a more aggressive type [20]. HPV 18 was not very prevalent and more frequently present in samples with a negative cytology. Follow up of these women would reveal the clinical relevance of these infections. The 12 HR-HPV cocktail accounted for the most common positive result and was present in 43% of ≥CIN2 cases. This finding is in accordance with other studies and deserves further exploration [19].

Regarding the semi-quantitative results given by both tests, RLU by HC2 and PCR cycle of detection (Ct) by cobas HPV Test, although higher viral loads were found statistically significant related to worse diagnosis, it does not seem very useful from a clinical point of view, due to a large distribution of values in each diagnostic category. However it is interesting that statistically significant differences were not found for HPV 16 or HPV 18 Ct values.

Beta-globin amplification is important in PCR based technology and our results showed that most cases had good quality DNA, only one being rejected. This finding confirms that liquid based cytology, in particular ThinPrep, is a good method for collecting and preserving cervical cells and DNA for molecular analyses.

Cases with concordant positive HC2 and cobas HPV Test results showed statistically significant higher viral loads (high HC2 RLUs/low Cts in cobas HPV Test) than cases with discordant results, explaining that low viral loads near cut off values account for a lower concordance between tests. A more detailed analysis of discordant results using other techniques may give more information on the small differences between tests, probably irrelevant from the clinical point of view since both tests shared a sensitivity >95% and a specificity >85%. Viral load by cobas HPV test was reproducible when comparing results of intralaboratory results, especially when Cts were under 35, however its clinical applicability cannot be drawn from this study.

In our setting, cobas HPV Test has proved to be a user-friendly system with high reproducibility and easy interpretation of results. We found some advantages in cobas compared to HC2. First of all there is no need for conversion when using PreservCyt samples. This procedure must be manually performed in badges of no more that 20 samples and takes approximately 2 hours. Moreover it is a possible cause of contamination and increases the risk of pipetting errors. The separate information on HPV types 16 and 18 may have clinical relevance regarding risk of persistence and progression, however our study was not designed to evaluate this objective and prospective studies would be more informative. We consider very valuable the DNA amplification of cellular beta-globin to assure good quality of the sample in the cobas HPV test. Actually, a negative result from a HC2 test could be due to the absence or low amount of cells on a sample while a cobas HPV test would yield an invalid result.

We feel confident that our results confirm a reliable clinical performance of cobas HPV Test allowing its introduction in cervical cancer screening protocols.

## References

[1] Muñoz N, Bosch FX, de Sanjose S, Herrero R, Castellsague X, et al (2003) Epidemiologic classification of human papillomavirus types associated with cervical cancer. N Engl J Med 348: 518–527. 12571259 10.1056/NEJMoa021641

[2] Bouvard V, Baan R, Straif K, Grosse Y, El Ghissassi F, et al (2009) Special report: Policy. A review of human carcinogens - Part B: biological agents. Lancet Oncol 10: 321–2. 19350698 10.1016/s1470-2045(09)70096-8

[3] Sherman ME, Lorincz AT, Scott DR, Wacholder S, Castle PE, et al (2003) Baseline cytology, human papillomavirus testing, and risk for cervical neoplasia: a 10-year cohort analysis. J Natl Cancer Inst 95: 46–52. 12509400 10.1093/jnci/95.1.46

[4] Cuzick J, Clavel C, Petry KU, Meijer CJ, Hoyer H, et al (2006) Overview of the European and North American studies on HPV testing in primary cervical cancer screening. Int J Cancer 119: 1095–101. 16586444 10.1002/ijc.21955

[5] Ronco G, Giorgi-Rossi P, Carozzi F, Dalla Palma P, Del Mistro A, et al (2006) New Technologies for Cervical Cancer Working Group. Human papillomavirus testing and liquid-based cytology in primary screening of women younger than 35 years: results at recruitment for a randomised controlled trial. Lancet Oncol 7: 547–55. 16814206 10.1016/S1470-2045(06)70731-8

[6] Mayrand MH, Duarte-Franco E, Rodrigues I, Walter SD, Hanley J, et al (2007) Canadian Cervical cancer Screening Trial Study Group. Human papillomavirus DNA versus Papanicolaou screening tests for cervical cancer. N Engl J Med 357: 1579–88. 17942871 10.1056/NEJMoa071430

[7] Dillner J, Rebolj M, Birembaut P, Petry KU, Szarewski A, et al (2008) Long term predictive values of cytology and human papillomavirus testing in cervical cancer screening: joint European cohort study. BMJ 337: a1754. 18852164 10.1136/bmj.a1754 PMC2658827

[8] Meijer CJLM, Berkhof J, Snijders PJF (2011) A new approach to cervical screening Lancet Oncol. Jul 12(7): 612–3. 10.1016/S1470-2045(11)70159-0 21684206

[9] Katki HA, Kinney WK, Fetterman B, Lorey T, Poitras N, et al (2011) Cervical cancer risk for women undergoing concurrent testing for human papillomavirus and cervical cytology: a population-based study in routine clinical practice. Lancet Oncol 12 (7): 667–72. 10.1016/S1470-2045(11)70145-0 PMC3272857 21684207

[10] Kinney W, Stoler MH, Castle PE (2010) Patient Safety and the Next Generation of HPV DNA Tests. Am J Clin Pathol 134: 193–9. 20660320 10.1309/AJCPRI8XPQUEAA3K

[11] Arbyn M, Sasieni P, Meijer CJ, Clavel C, Koliopoulos G, et al (2006) Clinical applications of HPV testing: A summary of meta-analyses. Vaccine 24: S78–S89. 10.1016/j.vaccine.2006.05.117 16950021

[12] Naucler P, Ryd W, Tornberg S, Strand A, Wadell G, et al (2007) Human papillomavirus and Papanicolaou tests to screen for cervical cancer. N Engl J Med 357: 1589–97. 17942872 10.1056/NEJMoa073204

[13] Bulkmans N, Berkhof J, Rozendaal L, van Kemenade F, Boeke A, et al (2007) Human papillomavirus DNA testing for the detection of cervical intraepithelial neoplasia grade 3 and cancer: 5-year followup of a randomised controlled implementation trial. Lancet 370: 1764–72. 17919718 10.1016/S0140-6736(07)61450-0

[14] Meijer CJ, Berkhof J, Castle PE, Hesselink AT, Franco EL, et al (2009) Guidelines for human papillomavirus DNA test requirements for primary cervical cancer screening in women 30 years and older. Int J Cancer 124: 516–20. 18973271 10.1002/ijc.24010 PMC2789446

[15] Heideman DAM, Hesselink AT, Berkhof J, van Kemenade F, Melchers WJG, et al (2011) Clinical validation of the cobas HPV Test for cervical screening purposes. J Clin Microbiol 49(11): 3983–5. 21880968 10.1128/JCM.05552-11 PMC3209101

[16] Solomon D, Davey D, Kurman R, Moriarty A, O’Connor D, et al (2002) for the Forum Group Members and the Bethesda 2001 Workshop The 2001 Bethesda System Terminology for reporting results of cervical cytology JAMA. 287(16): 2114–19. 10.1001/jama.287.16.2114 11966386

[17] Schiffman M, Solomon D (2003) Finding to date from the ASC-US-LSIL Triage Study (ALTS). Arch Pathol Lab Med (127) 946–949. 10.5858/2003-127-946-FTDFTA 12873166

[18] Arbyn M, Ronco G, Meijer CJLM, Naucler P (2009) Trials comparing cytology with human papillomavirus screening. Lancet Oncol 10: 935–6. 19796748 10.1016/S1470-2045(09)70296-7

[19] Stoler MH, Wright TC Jr, Sharma A, Apple R, Gutekunst K, et al (2011) High-risk human papillomavirus testing in women with ASC-US cytology: results from the ATHENA HPV study. Am J Clin Pathol. 135: 468–75. 10.1309/AJCPZ5JY6FCVNMOT 21350104

[20] Khan MJ, Castle PE, Lorincz AT, Wacholder S, Sherman M, et al (2005) The elevated 10-year risk of cervical precancer and cancer in women with Human Papillomavirus (HPV) Type 16 or 18 and the possible utility of type-specific HPV testing in clinical practice. J Natl Cancer Inst 97: 1072–9. 16030305 10.1093/jnci/dji187

