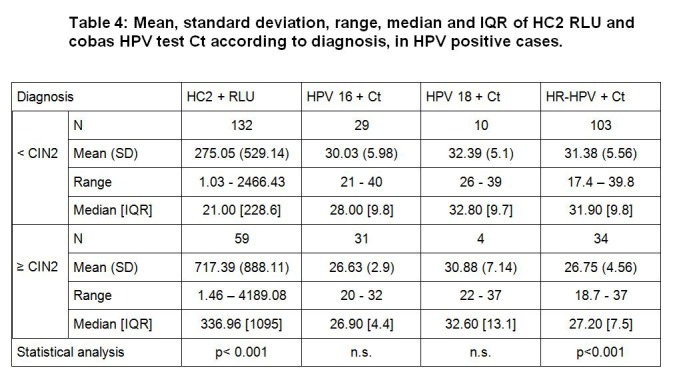# Correction: HPV Testing by cobas HPV Test in a Population from Catalonia

**DOI:** 10.1371/annotation/e228bdf4-d943-4d44-8e0f-d441dfa80260

**Published:** 2013-10-25

**Authors:** Belén Lloveras, Silvia Gomez, Francesc Alameda, Beatriz Bellosillo, Sergi Mojal, Mercè Muset, Manuel Parra, José Carlos Palomares, Sergi Serrano

In Table 4, the first line of the table is shifted to the right. The first row should read Diagnosis, HC2 + RLU, HPV 16 + Ct, HPV 18 + Ct, HR-HPV + Ct. Please see the corrected Table 4 here: 

**Figure:**